# Expanding the scope of human immunology in the *Journal of Human Immunity*

**DOI:** 10.70962/jhi.20260012

**Published:** 2026-02-13

**Authors:** Petter Brodin, JHI associate and consulting editors, Dusan Bogunovic, Andy Gennery, Elena Hsieh, Isabelle Meyts, Tomohiro Morio, Cecilia Poli, Anne Puel, Neil Romberg, Vijay Sankaran, Helen Su, Stuart Tangye, Stuart Turvey, Shen-Ying Zhang, Yanick Crow, Josh Milner, Luigi Notarangelo, JHI society editors, Amita Aggarwal, Hamoud Al-Mousa, Ahmed Aziz Bousfiha, Sophie Hambleton, Fabian Hauck, Carrie L. Lucas, Cindy S. Ma, Elissaveta Naumova, Satoshi Okada, Carolina Prando, Amit Rawat, Nima Rezaei, Andrew L. Snow, Xiaochuan Wang, Megan Cooper, Jean-Laurent Casanova

**Affiliations:** 1 Department of Women’s and Children’s Health, Karolinska Institutet, Solna, Sweden; 2 Department of Immunology and Inflammation, Imperial College London, London, UK; 3 Medical Research Council Laboratory of Medical Sciences, Imperial College Hammersmith Campus, London, UK; 4 Division of Rheumatology/Immunology, Department of Pediatrics, Washington University in St. Louis, St. Louis, MO, USA; 5 St Giles Laboratory of Human Genetics of Infectious Diseases, Rockefeller Branch, The Rockefeller University, New York, NY, USA; 6 Howard Hughes Medical Institute, New York, NY, USA; 7 Laboratory of Human Genetics of Infectious Diseases, Necker Branch, INSERM, Necker Hospital for Sick Children, Paris, France; 8 Imagine Institute, Paris Cité University, Paris, France; 9 Pediatric Hematology and Immunology Unit, Necker Hospital for Sick Children, Paris, France

## Abstract

The *Journal of Human Immunity (JHI)* publishes molecular, cellular, and clinical studies of patients with inborn errors of immunity, or their phenocopies, including autoimmune and somatic disorders. A central tenet in the field is that the current range of genetic immunological disorders is only the tip of the iceberg, given the wide range of conditions and populations, and countless patients not yet studied from this angle. Systematic research into causal monogenic lesions is appropriate and likely to be informative in many infectious, allergic, inflammatory, autoimmune, and malignant disorders. Rare or even private genetic etiologies may be of heuristic value, revealing physiological mechanisms disrupted by other, more common genetic or other causes in other patients. In this context, the journal welcomes all immunological studies in line with this vision, in which molecular, cellular, or clinical abnormalities in individuals are seen as candidate phenotypes, potentially driven by inborn errors of immunity—monogenic or otherwise—worthy of genetic investigation.

## Teaser

A broader scope for the *Journal of Human Immunity*.

The field of human immunology is gaining momentum. An increasing number of researchers are studying healthy individuals, patients, or both, providing a long-awaited holistic complement to the reductionist animal models that have historically dominated the field of immunology (1). This transition has been facilitated by technological advances making it possible to extract more information from a single blood or tissue sample (2), and by the realization that most animal models neither fully nor faithfully reproduce the diversity of human diseases and their genetic and environmental determinants (3). Historically, inbred animals were studied to control for genetic background, but the enormous genetic diversity of humans has become an asset since the first human genome sequence became available and with the advent of next-generation sequencing, making it possible to sequence entire individual genomes both swiftly and cheaply. Moreover, while experiments in animals were appreciated for their rigor, experiments of nature in humans provide added value in not being biased by the experimentalist (4).

The careful observation and description of patients with rare or even private phenotypes have almost always preceded the discovery and characterization of underlying inborn errors of immunity (IEIs). Such studies paved the way for the discovery of monogenic etiologies of more common infectious, autoimmune, autoinflammatory, malignant, or allergic phenotypes (5). At odds with what is widely believed, single-gene genotypes involving common variants can also underlie common conditions. An example is provided by homozygosity for P1004A TYK2, which underlies about 1% of European cases of tuberculosis (6). The field has also evolved in appreciating both Mendelian (fully penetrant) and non-Mendelian monogenic disorders (incompletely penetrant), with the recent elucidation of several mechanisms of incomplete penetrance, including adaptive immunity, genetic modifiers, and random monoallelic expression (7). Another recent development is the description of genetic modifiers that influence the expressivity of monogenic diseases (8, 9, 10). These advances help explain the phenotypic variability of presumed monogenic disorders. Historically, most of the chromosomal abnormalities underlying immunological phenotypes, such as trisomy 21 (Down’s syndrome), have not been associated with the field of IEI. However, such an association has been reported for others, such as 22q11.2 deletion syndrome (DiGeorge syndrome) (11).

The molecular causes of >500 IEIs have now been described, corresponding to ∼10% of all known genes for which single-gene lesions underlie human phenotypes (total *n* = 5,041, https://www.omim.org/). These genes have provided invaluable information about the role of specific molecules, pathways, and cells in a growing number of conditions (5). The large proportion of known disease-causing genes associated with immunological disease manifestations—whether infectious, inflammatory, allergic, autoimmune, or tumoral—illustrates the strong evolutionary pressures exerted by microorganisms on humans, driving the evolution of robust defense mechanisms (12).

As a journal co-owned by the International Alliance of Primary Immunodeficiency Societies (IAPIDS) and its seven constituent societies and affiliated with other societies in the field of IEI, *JHI* naturally focuses on the publication of molecular, cellular, or clinical studies of patients with IEIs (4). These studies are, almost by definition, dependent on knowledge about rare clinical and immunological phenotypes (e.g., autosomal dominant hyper-IgE syndrome), causal genotypes (e.g., dominant-negative mutations of *STAT3*), or both.

However, we believe that the *JHI* should also welcome other studies that consider immunological conditions, ranging from unique to common illnesses, to be potentially monogenic or more rarely chromosomal, at least in some patients, despite the absence of a well-known genotype or phenotype. Reports of unique or rare phenotypes as being potentially due to an IEI are not new and date right back to the birth of this field. However, the *JHI* intends to push the boundaries of this approach, in which patients with common conditions are considered through the prism of IEI, much further (13). Reports of relatively common immunological and clinical manifestations as candidate phenotypes for IEI pave the way for the discovery of underlying single-gene IEIs, or causal genetic lesions more generally.

However, not all patients carry causal genetic lesions just waiting to be discovered. In many cases, a combination of careful phenotypic description and genetic investigation fails to identify a known or new IEI. This may be due to low penetrance of a genetic lesion, the involvement of another genetic architecture, an autoimmune or somatic genetic phenocopy, or even a nongenetic cause of disease. Nevertheless, this human-centered approach, in which the null hypothesis is that a patient’s phenotype is driven by a monogenic lesion, can offer important insights that help to unravel pathogenesis, provided the starting point of the analysis is a careful description of the phenotype. Common disease mechanisms may be revealed by rare or even single patients with an IEI (14). For example, pathogenic autoantibodies neutralizing type I IFN were reported in only one patient between 1981 and 1984 (15), but have recently been widely described as phenocopies of IEIs underlying life-threatening COVID-19 (16), influenza (17), and a growing number of forms of arboviral encephalitis (18, 19) in significant proportions of patients. Another condition, idiopathic pulmonary alveolar proteinosis, is an immune dysregulation in the lung caused by the production of neutralizing autoantibodies against GM-CSF (20). Similar clinical phenotypes are seen in patients with inherited GM-CSF receptor deficiency (21). These examples, discovered through the careful assessment of patients with rare clinical presentations, illustrate the potential of clinical studies to be informative, even in the absence of monogenic causes. Secondary immunodeficiencies caused by viruses or, more commonly, immunosuppressive drugs are of considerable clinical importance, but they remain difficult to disentangle from the underlying condition and its effect on immunity. In this light, we would argue that studies on secondary immunodeficiencies would be better published in journals other than *JHI*, unless a clear connection can be made with IEI. For example, a mechanistic explanation for an infectious disease striking HIV-infected individuals, inspired by the study of IEI, would probably be considered appropriate for the journal (22).

Therefore, *JHI* welcomes clinical and immunological studies of human patients, especially those with unusual presentations (rare or common), across diverse clinical specialties, with or without familial aggregation, when there are reasons to consider that the patients may display phenotypes of genetic origin or immunological phenocopies of such phenotypes. Known examples include common granulomatous diseases, such as Crohn’s disease, the study of which has been inspired by that of the rarer monogenic Blau syndrome (23). Likewise, the discovery of ultrarare inborn errors of IL-10 in patients with early-onset inflammatory bowel disease (IBD) led to the discovery of neutralizing auto-Abs against IL-10 (24). Such autoantibodies could perhaps explain IBD in many more patients. Chromosomal aberrations, such as 22q11.2 deletion syndrome (DiGeorge syndrome), are well known in the field of primary immunodeficiency (11); increasing attention has recently focused on the variable immune dysregulation associated with trisomy 21 (Down’s syndrome) (25). This highlights the need for more detailed investigations of the immune system also in other syndromes caused by chromosomal aberrations. The editorial board of *JHI* would be delighted to receive articles resulting from such studies.

In patients with excessive type 2 immunity, atypical atopic manifestations or a lack of response to treatment may suggest an underlying monogenic cause (26). Similarly, in patients with rheumatologic presentations, a lack of response to standard immunomodulation or the presence of unusual features should be recognized and reported in the absence of identified molecular causes (27). Even in the absence of monogenic lesions, or of any other type of causal genetic lesion, such studies would be of interest to the readership of *JHI*.

This rationale can be extended further: even populations of patients diagnosed with common diseases might include rare cases with underlying monogenic causes of a disease with an overlapping presentation but potentially different responses to treatment, familial aggregation and age at presentation, or cases displaying none of these “red flags.” For the purposes of illustration, a recent genetic analysis of individuals within the UK Biobank and large clinical trial cohorts of patients with multiple sclerosis, IBD, and atopic dermatitis (or eczema) identified several known monogenic disease-causing variants among these individuals with common diseases (28). These observations are consistent with the idea that diseases are merely just “labels,” helpful at the bedside for a certain period, until further medical progress breaks them down into smaller categories. This is best illustrated by “fever,” a term that has been broken down into thousands of conditions. In this light, the asymptote is that every patient is unique, in which case the dichotomy between rare and common disease disappears (4).

In conclusion, the editorial board of *JHI* is committed to publishing the best manuscripts related to IEI, in accordance with its mandate from IAPIDS. At the same time, the editorial board of *JHI* welcomes manuscripts from researchers outside the field of IEI focusing on careful descriptions of molecular, cellular, immunological, or clinical manifestations that can be considered as human immunological phenotypes—including physiological and pathological phenotypes—possibly due to a monogenic or chromosomal cause of disease (i.e., any clearly causal genetic lesion, or its autoimmune or somatic determinant). Such phenotypic descriptions are important and can help guide future research in human immunology, beyond strict genotype-to-phenotype studies. As a guiding principle for the identification of high-quality studies of interest to *JHI*, we are inspired by the words and works of the legendary human immunologist Henry Kunkel, who exemplified “patient-based basic research” at its best (https://www.henrykunkelsociety.org/).

## Supplementary Material

Table S1 provides the list of affiliations for the associate and consulting editors.

Table S2 provides the list of affiliations for the society editors.
