# Supplementary material for: Expanding the scope of human immunology in the Journal of Human Immunity
Source: J Hum Immun. 2026 Feb 13;2(2):e20260012. doi: 10.70962/jhi.20260012 (PMC12903872; doi:10.70962/jhi.20260012)
Supplement: Table S1 — provides the list of affiliations for the associate and consulting editors. [file jhi_20260012_tables1.docx]

**JHI associate and consulting editors:**

### Dusan Bogunovic^1^, Andy Gennery^2^, Elena Hsieh^3^, Isabelle Meyts^4^, Tomohiro Morio^5^, Cecilia Poli^6^, Anne Puel^7^, Neil Romberg^8^, Vijay Sankaran^9^, Helen Su^10^, Stuart Tangye^11^, Stuart Turvey^12^, Shen-Ying Zhang ^7^, Yanick Crow^13^, Josh Milner^14^, Luigi Notarangelo^15^

^1^Department of Pediatrics, Center for Genetic Errors of Immunity, Columbia University, New York, NY, USA

^2^Department of Pediatric Immunology, Great North Children’s Hospital, Royal Victoria Infirmary, Newcastle upon Tyne, UK

^3^Department of Pediatrics, Section of Allergy and Immunology, Department of Immunology and Microbiology, University of Colorado School of Medicine, Aurora, CO USA.

^4^Department of Pediatrics, University Hospitals Leuven; KU Leuven, Department of Microbiology, Immunology and Transplantation; Laboratory for Inborn Errors of Immunity, KU Leuven, Leuven, Belgium.

^5^Department of Pediatrics and Developmental Biology Institute of Science Tokyo Tokyo Japan

^6^Program of Immunogenetics and Translational Immunology, Facultad de Medicina, Clínica Alemana Universidad del Desarrollo, Santiago, Chile; Hospital de niños Dr. Roberto del Rio, Santiago, Chile.

^7^Laboratory of Human Genetics of Infectious Diseases, Necker Branch, INSERM U1163, Necker Hospital for Sick Children, Paris, France; Paris Cité University, Imagine Institute, Paris, France; St. Giles Laboratory of Human Genetics of Infectious Diseases, Rockefeller Branch, Rockefeller University, New York, NY, USA.

^8^Division of Immunology and Allergy, Children's Hospital of Philadelphia, Philadelphia, Pa; Department of Pediatrics, Perelman School of Medicine, University of Pennsylvania, Philadelphia, PA, USA.

^9^Division of Hematology/Oncology, Boston Children's Hospital, Harvard Medical School, Boston, MA, USA; Department of Pediatric Oncology, Dana-Farber Cancer Institute, Harvard Medical School, Boston, MA, USA.

^10^Human Immunological Diseases Section, Laboratory of Clinical Immunology and Microbiology, Intramural Research Program, National Institute of Allergy and Infectious Diseases, National Institutes of Health, Bethesda, MD, USA.

^11^Garvan Institute of Medical Research, Darlinghurst, NSW, Australia; St Vincent’s Clinical School, Faculty of Medicine, UNSW Sydney, NSW, Australia.

^12^BC Children’s Hospital, The University of British Columbia, Vancouver, Canada.

^13^Laboratory of Neurogenetics and Neuroinflammation, Imagine Institute, Paris, France; MRC Human Genetics Unit, Institute of Genetics and Cancer, University of Edinburgh, Edinburgh, UK.

^14^Division of Pediatric Allergy, Immunology and Rheumatology, New York-Presbyterian/ Columbia University Irving Medical Center, New York, NY.

^15^Laboratory of Clinical Immunology and Microbiology, National Institute of Allergy and Infectious Diseases, NIH, Bethesda, Maryland, USA.
