# Supplementary material for: Expanding the scope of human immunology in the Journal of Human Immunity
Source: J Hum Immun. 2026 Feb 13;2(2):e20260012. doi: 10.70962/jhi.20260012 (PMC12903872; doi:10.70962/jhi.20260012)
Supplement: Table S2 — provides the list of affiliations for the society editors. [file jhi_20260012_tables2.docx]

**JHI society editors:**

Amita Aggarwal^1^, Hamoud Al-Mousa^2^, Ahmed Aziz Bousfiha^3^, Sophie Hambleton^4^, Fabian Hauck^5^, Carrie L. Lucas^6^, Cindy S. Ma^7^, Elissaveta Naumova^8^, Satoshi Okada^9^, Carolina Prando^10^, Amit Rawat^11^, Nima Rezaei^12^, Andrew L. Snow^13^, Xiaochuan Wang^14^

^1^ Dept. of Clinical Immunology and Rheumatology, Sanjay Gandhi Postgraduate Institute of Medical Sciences, Lucknow, India.

^2^ Dept. of Pediatrics, King Faisal Specialist Hospital & Research Center; College of Medicine, Alfaisal University, Riyadh, Saudi Arabia.

^3^Laboratory of Clinical Immunology, Infection, and Autoimmunity, Faculty of Medicine and Pharmacy of Casablanca, Hassan II University, Casablanca, Morocco.

^4^Translational and Clinical Research Institute, Faculty of Medical Sciences, Newcastle University, Newcastle upon Tyne, UK.

^5^Dr. von Hauner Children's Hospital, Ludwig-Maximilians-University, Munich, Germany.

^6^Immunobiology Department, Yale University School of Medicine, New Haven, CT, USA.

^7^Garvan Institute of Medical Research; Sydney Medical School, University of Sydney, Sydney, New South Wales, Australia.

^8^Medical Faculty, Department of Clinical Immunology; University Hospital Alexandrovska, Clinic of Clinical Immunology and Stem Cell Bank, Sofia, Bulgaria.

^9^ Dept. of Pediatrics, Hiroshima University Graduate School of Biomedical and Health Sciences, Hiroshima, Japan.

^10^Hospital Pequeno Príncipe, Água Verde, Curitiba, PR, Brazil.

^11^Allergy and Immunology Unit, Department of Pediatrics, Advanced Pediatrics Center, Postgraduate Institute of Medical Education and Research, Chandigarh, India.

^12^Tehran University of Medical Sciences, Tehran, Iran.

^13^ Dept. of Pharmacology and Molecular Therapeutics, Uniformed Services University of the Health Sciences, Bethesda, MD, USA.

^14^ Dept. of Clinical Immunology, Children's Hospital of Fudan University, National Children's Medical Center; Shanghai Institute of Infectious Disease and Biosecurity, Shanghai, China.
